# Root-Related Genes in Crops and Their Application under Drought Stress Resistance—A Review

**DOI:** 10.3390/ijms231911477

**Published:** 2022-09-29

**Authors:** Tianyuan Qin, Ali Kazim, Yihao Wang, Dormatey Richard, Panfeng Yao, Zhenzhen Bi, Yuhui Liu, Chao Sun, Jiangping Bai

**Affiliations:** 1 State Key Laboratory of Aridland Crop Science, College of Agronomy, Gansu Agricultural University, Lanzhou 730070, China; 2 National Institute for Genomics and Advanced Biotechnology, National Agricultural Research Centre, Park Road, Islamabad 45500, Pakistan

**Keywords:** root architecture, root developmental genes, stress resistance

## Abstract

Crop growth and development are frequently affected by biotic and abiotic stresses. The adaptation of crops to stress is mostly achieved by regulating specific genes. The root system is the primary organ for nutrient and water uptake, and has an important role in drought stress response. The improvement of stress tolerance to increase crop yield potential and yield stability is a traditional goal of breeders in cultivar development using integrated breeding methods. An improved understanding of genes that control root development will enable the formulation of strategies to incorporate stress-tolerant genes into breeding for complex agronomic traits and provide opportunities for developing stress-tolerant germplasm. We screened the genes associated with root growth and development from diverse plants including *Arabidopsis*, rice, maize, pepper and tomato. This paper provides a theoretical basis for the application of root-related genes in molecular breeding to achieve crop drought tolerance by the improvement of root architecture.

## 1. Introduction

Global warming and water scarcity attributable to climate change have caused an urgent global food security challenge. One third of the world’s arable land is undersupplied with water, while other arable land is frequently affected by periodic or unpredictable droughts that have resulted in yield losses of up to approximately USD 30 billion in the last decade [1,2]. As the global population continues to grow (and is expected to increase to approximately 10 billion people by 2050 [3]), the demand for water for agriculture and the reduced availability of freshwater will further exacerbate the impact of droughts on agriculture [4]. Therefore, studying the mechanisms by which plants can sustain growth during droughts, and the exploration of strategies to improve plant survivability may provide solutions to future food security problems [5,6,7]. In China, sustainable agricultural development is severely affected by the large proportion of arid and semi-arid regions, high water consumption in agriculture, and low irrigation water-use efficiency. Irrigation and improvement of the water-holding capacity of soil are effective agronomic measures to compensate for low rainfall, but selection and breeding of drought-tolerant plant cultivars is an additional effective strategy to improve the water-use efficiency of crops. Therefore, plant breeders are targeting leaf growth and transpiration traits to improve plant reproductive phenology under water limitation [8]. However, water uptake and root phenotypic traits, which are the most important factors affecting plant water uptake, have not been adequately addressed by breeders mainly because of the high variability of root systems in relation to the environment and the difficulty of in situ monitoring of root systems. Therefore, understanding the response of root cells to water deficit is crucial for continued sustainable agricultural production [9,10,11].

Potato (*Solanum tuberosum* L.) is an important tuberous crop and is among the four largest crops in the world. Global potato production is currently about 350 billion kg per year and is increasing annually [12,13]. China is the largest potato producer in the world. Potato-growing areas in China are mainly located in the northwest, western Inner Mongolia, the northeast, and other arid and semi-arid regions with an average annual precipitation less than 500 mm. Stress from prolonged or seasonal drought can severely affect potato plant growth, tuber yield, and marketability. Especially in the tuber growth stage, drought stress can cause significant yield loss or even crop failure [14,15]. Therefore, research on the drought tolerance of potato is essential to ensure productivity under global climate change.

In this review, we compiled 217 published genes associated with root development from diverse plant species, including *Arabidopsis*, rice, maize, pepper and tomato. Our future aim is to further validate the functions of these genes in potato and apply them in breeding programs to improve potato drought tolerance.

## 2. Root Traits Associated with Drought Stress Tolerance

The root system plays an essential role in the plant life cycle and forms a complex structure through growth and branching to fulfil its primary functions of anchoring the plant in the soil and absorbing water and nutrients [16,17]. Important structural characteristics of the root system include the length of the primary root, the density of the secondary roots, and the gravitropic set-point angle of the root, all of which are mostly regulated by plant hormones [18,19]. In rice and *Arabidopsis*, a deep-penetrating root system and positively geotropic root growth are the predominant traits that govern drought adaptation, as both phenotypes facilitate water uptake from deeper layers of the soil and help to ensure that normal plant activities are maintained [20].

In addition to the root architecture, water uptake depends on the intrinsic water-transport capacity of the root system. Normally, water absorbed by the root is radially transferred to the central stele and transported in the xylem to the aboveground organs, where water channel proteins (termed aquaporins) play a vital role in intercellular water transport. The root system responds to changes in soil water availability at the cellular and root architecture levels, and the root system cell ecological niche, phloem tissue, and vascular system coordinate to respond to adverse stresses [21]. When a plant perceives water deficit, the architecture of the root system is subjected to morphological changes through cell division, elongation, and differentiation at the root tip to improve the water uptake capacity. Root system architecture is associated with the distribution and depth of the soil water layer. The deeper the root system, the smaller the branching angle of the root tip, and the more efficiently the root system can absorb water from deeper soil layers [22]. Lignification and suberization of specific walls of the endodermal cells play an important role in water transport in the xylem. The physiological water balance of the plant is actively maintained by water transport in the xylem from the roots to the aboveground organs and photosynthetic assimilate transport in the phloem from the shoots to the root system. The transport process may also affect the drought tolerance of the plant [23].

In general, many traits can be used to screen plant roots for drought tolerance, including the germination capacity of the plant in soils with different osmotic potentials, the depth, width, and thickness of roots in the soil, the starch hydrolysis status of the root crown, and the proline accumulation capacity of the root system under drought, as well as antioxidant enzyme activities and root vitality [24,25,26,27,28]. In addition, morphological variables, such as root growth and yield indicators, can be used to assess drought resistance in crops [29,30]. Therefore, a comprehensive assessment combining the above indicators will more realistically reflect the actual drought resistance of a crop species. Since the root system is the main organ that allows plants to absorb water, it is necessary to comprehensively understand the genes related to root growth and development.

## 3. Current Status of Research on Genes Associated with Root Growth and Development

Distribution in the soil and degree of development of the root system are directly related to the growth and development of aerial shoots. Most plants depend on the root system to supply the water and minerals essential for the growth and maintenance of the aboveground organs. A balance is established between the development of the branches and leaves and the development of the root system [31,32]. The development of the root system is much greater than that of the aboveground parts, and the total area of the root system in contact with the soil often exceeds the area of the stem and leaves by a factor of 6–16 [33]. The spatial configuration of the root system can be generally divided into crown roots, primary roots, lateral roots, and adventitious roots. Root elongation, water and nutrient uptake, tissue differentiation, and response to gravity and light all occur in the root tip, which consists of the root cap, meristematic zone, elongation zone, and maturation zone. The root cap protects the growing point of the root tip from friction and damage from the soil and is the primary gravity-sensing site. The epidermal cells of the maturation zone project outward to form root hairs. As root hairs are shed and the root tip grows, root hairs develop from newly generated cells; thus, a root hair zone is always maintained at the root tip, which is the most active portion of the root for absorbing water and inorganic salts [34,35,36].

**Figure 1:**
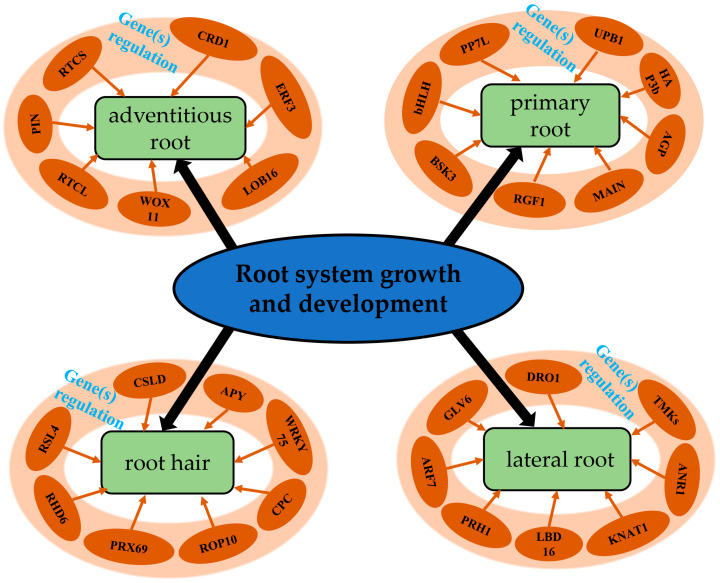
Major genes related to root system growth and development.

We conducted a literature search and identified 217 published genes associated with different root development traits (Table 1) to investigate the important functions of plant root systems. The major genes that represent root growth and development are shown in Figure 1.

### 3.1. Genes Associated with Adventitious Root Growth and Development

Adventitious roots are an important component of potato root system architecture (RSA); thus, understanding adventitious root development may be useful to improve potato yield and optimize the potential of agricultural land use [37]. Roots are not only the principal organs for water and nutrient uptake in plants, but they also respond to environmental and plant–soil microbial interactions [38,39]. Rice, an important food crop, has a fibrous-rooted system. The subsequent growth of the rice root system depends on adventitious roots that continuously emerge from the germinal sheath or the base of stem nodes. Scientists such as Zhou Daoxiu and Zhao Yu have been studying rice epigenetics and the mechanisms that control the development of adventitious roots in rice since as early as 2009. They identified the *WUSCHEL-Related Homeobox 11* (*WOX11*) gene from the WUSCHEL-related homeobox domain family, an important regulator of adventitious root development in rice. *WOX11* is specifically expressed in the meristematic zone of adventitious roots in rice after elongation. Overexpression of *WOX11* not only increases the number of adventitious roots but can also give rise to ectopic roots on the rice stems and at the base of the flower. Reduced expression or the complete loss-of-function of this gene results in a dramatic reduction in the adventitious root phenotype. Further studies revealed that *WOX11* directly represses the expression of *Ribonucleotide Reductase 2* (*RR2*), encoding an A-type cytokinin response factor that participates in the development of adventitious roots in rice [40]. *WOX11* and the AP2-like transcription factor *ERF3* (*EUKARYOTIC RELEASE FACTOR 3*) precisely regulate the expression of *RR2* in a reciprocal manner to control the initiation and elongation of adventitious roots in rice [41]. The genes *ZmRTCS* and *ZmRTCL* are important regulators of adventitious root formation in maize and both RTCS (rootless concerning crown and seminal roots) and RTCL (RTCS-like) proteins bind to the LBD (lateral organ boundaries domain) downstream promoter response element *ARF34* and function as transcription factors. Mutation of *RTCL* leads to the early growth and developmental arrest of adventitious roots in maize, and *RTCS* regulates transcriptional expression of the *RTCL* gene in the maize root, demonstrating the synergistic roles of *RTCS* and *RTCL* in adventitious root formation [42].

In *Arabidopsis*, *PIN-FORMED* (*PIN*) polarity regulators have been studied by mutagenizing the *PIN2*:*PIN1-HA*; *pin2* strain and identifying the *regulator of PIN polarity 12* (*repp12*) mutant, which restored the gravitropic growth phenotype of the *Arabidopsis* adventitious root system [43,44]. Similarly, a study on rice showed that *OsPIN* is expressed in vascular tissue and root primordia in a manner similar to *Arabidopsis AtPIN1*. In transgenic rice, in which *OsPIN1* was silenced by RNA interference (RNAi), root emergence and development are significantly inhibited. Overexpression or suppression of *OsPIN1* expression by transgenic methods result in significant changes in the number of tillers and the root-to-shoot ratio, suggesting that *OsPIN1* is important in rice root growth and tillering [45]. In addition, a novel regulator of adventitious root development in rice, *CROWN ROOT DEFECT 1* (*CRD1*), was identified by screening a rice mutant library. Zhu et al. [46] revealed that *CRD1* affects adventitious root development in rice by regulating the development of adventitious root primordia. Small RNA sequencing of wild type and the mutant revealed that *CRD1* can regulate miR156 levels, thereby modulating adventitious root development. In addition, the authors showed that *CRD1* can maintain miRNA stability and they demonstrated its essential role in adventitious root development in indica and japonica rice cultivars. Several mutant tomato lines have been generated by CRISPR/Cas9-mediated editing of a Cas9/single-guide RNA construct targeting the second exon of *CCD8* (^CCD8^Cas9). The T_1_ plants of the ^CCD8^Cas9 mutant exhibited several morphological changes including dwarfism and formation of excessive adventitious roots [47]. Among four potato cultivars differing in earliness, drought reduced the maximum dry mass of roots and the total length of stolons, but increased stolon number. The number of adventitious roots on stolons was decreased under drought stress and was negatively correlated with the root dry mass of plants. Fresh tuber yield was significantly correlated with root dry mass in the field, and the drought tolerance index was significantly correlated with root depth in the field [48,49].

### 3.2. Genes Associated with Primary Root Growth and Development

The primary root, which develops from the radicle, has a strong growth ability and can grow to a depth of 2–3 m. In a study of *Arabidopsis*, Jia et al. [50] used genome-wide association analysis to identify genes associated with changes in primary root length. The authors identified *BRASSINOSTEROID-SIGNALING KINASE 3* (*BSK3*) as the main gene effecting primary root length. In addition, the basic helix-loop-helix (bHLH) transcription factor *UPBEAT1* (*UPB1*) regulates the balance between cell proliferation and differentiation by directly controlling peroxidase expression [50]. The differential localization of *UPB1* in transcriptional and translational reporter gene lines of *Arabidopsis* suggests that deletion mutants of *UPB1* lead to impaired growth of the primary root. The transcriptional regulator may also function as an intercellular signaling molecule and provide a direct transcriptional link between the distribution of reactive oxygen species (ROS) and the proliferation state of root apical cells [51]. In *Arabidopsis*, overexpression of *HOOK-ASSOCIATED PROTEIN 3B* (*HAP3b*) promotes elongation of the primary root. Root cells overexpressing *HAP3b* elongate faster than wild-type root cells, and HAP3b is specifically expressed in the apical region and promotes apical cell division and elongation [52].

Two heterologous genes, *MAINTENANCE OF MERISTEMS* (*MAIN*) and *MAINTENANCE OF MERISTEMS LIKE 1* (*MAIL1*), in *Arabidopsis* encode a conserved retrotransposon-associated mobile plant domain essential for primary root development [53]. Loss-of-function of *MAIN* or *MAIL1* results in the release of heterochromatin, reduced cohesion of heterochromatin around metaphase, cell death of meristematic tissue, and growth arrest shortly after primary root emergence. In addition, loss-of-function of *PROTEIN PHOSPHATASE 7-LIKE* (*PP7L*) results in the same root growth phenotype as the loss-of-function *main* or *mail1* mutants, with the *PP7L* mutant showing an incipient root growth arrest phenotype. A double-mutation analysis confirmed that the three genes, *MAIN*, *MAIL*, and *PP7L* act in the same molecular pathway [54]. *ROOT MERISTEM GROWTH FACTOR 1* (*RGF1*), a secreted peptide hormone, assists/regulates phloem development in *Arabidopsis* primordial roots. *RGF1* regulates root phloem tissue activity mainly through two downstream transcription factors, *PLETHORA 1* (*PLT1*) and *PLT2*. Two independent *rgi1 rgi2 rgi3 rgi4 rgi5* quintuple mutants exhibit a consistent short primary root phenotype. Expression of *PLT1* and *PLT2* is barely detectable in the quintuple mutants. Ectopic expression in the quintuple mutant of *PLT2* driven by the *RGF1 INSENSITIVE 2* (*RGI2*) promoter largely reverses the primary root meristem defects. Exogenous administration of *RGF1* rapidly and simultaneously induces phosphorylation and ubiquitination of *RGI1*, which enables *RGI1* to recognize and transduce peptide signals from *RGF1*. Thus, RGIs function as receptors for *RGF1* and regulate meristem development in *Arabidopsis* primordial roots [55].

In rice, two T-DNA insertion mutant strains of *ROOT LENGTH REGULATOR 4* (*OsRLR4*) developed primary roots longer than those of the wild type, whereas the opposite was true for the overexpression strains. Inukai et al. [56] characterized five recessive mutants in rice, *BRX65*, *BRX117*, *BRX430*, *BRX448* and *crl2* mutants, to analyze the genetic mechanisms controlling root elongation. The mutants, which showed a short root length phenotype, were genetically defined as *reduced root length* (*rrl*). The first *rrl* locus was designated as *RRL1* (BRX65), the other locus as *RRL2*, and the alleles were designated as *RRL2-1* (BRX117), *RRL2-2* (BRX430), and *RRL2-3* (BRX448). In the *rrl1* mutant, only the mature cortical cells were significantly shorter than in the wild type, whereas the roots of the *rrl2-1* mutant had significantly shorter cell length, apical meristem size, and cell flux than the wild type. However, mature cortical cell length, apical meristem size, and cell flux were significantly higher in roots of the *crl2* mutant than in the wild type. The *rrl1 crl2* double mutant had a mature bark cell length intermediate between the parental single mutants, whereas the *rrl2-1 crl2* double mutant had cell length, meristem size, and cell flux intermediate between the parental single mutants. These results suggest that opposing effects of these genes determine the extent of primary root growth and development in rice [57]. In addition, the *OsAGP* gene in rice encodes a protein with a structure similar to that predicted by *ArfGAP*. The purified OsAGP-GST fusion protein is able to stimulate the GTPase activity of rice Arf. In addition, *OsAGP* is able to repair the defective vesicular translocation in yeast cells of the *gcs1*∆*glo3*∆ double mutant. Transgenic *Arabidopsis* constitutively expressing *OsAGP* shows reduced apical dominance and shortened primary roots. These results suggest that rice ARF-GAP may be involved in regulating plant root growth and development [58].

A receptor gene for Glu, designated as *GLR3*, has a T-DNA insertion that results in a mutant phenotype of shortened rice primary roots. Histological and DNA synthesis analyses showed that the activity of the mutant root meristem is impaired and associated with enhanced programmed cell death. Thus, in rice, *GLR3* is essential for the maintenance of cell division and cell survival in the early root tip meristem tissue of seedlings [59]. Similarly, a short postembryonic root mutant of rice, designated as *OsSPR1*, has shorter postembryonic roots and encodes a novel mitochondrial protein with an armadillo-like repeat structural domain. Complementation experiments with the *spr1* mutant have confirmed the involvement of the *OsSPR1* mutant in the elongation growth of rice postembryonic roots [60]. The rice mutant *Osglu3-1*, with a short root phenotype caused by a point mutation in *OsGLU3*, which encodes a membrane-bound protein, was isolated and identified from a rice library mutagenized with ethyl methanesulfonate (EMS). The *Osglu3-1* mutant has less crystalline cellulose in the root cell wall, shorter root cells, and less root meristem tissue. *OsGLU3* is widely expressed in various tissues, and especially strongly in the roots, and regulates root elongation by changing the cell wall cellulose content [61].

Mapping-based cloning revealed that an EMS-mutagenized rice mutant with short roots was caused by a point mutation, causing premature termination during protein synthesis in an intron of *OsDGL1*. *OsDGL1* is a direct homolog of *Arabidopsis DGL1* and yeast *WBP1* and is involved in N-glycosylation in eukaryotes. The *Osdgl1* rice mutant exhibits alternations in the polysaccharides of the root cell wall matrix, resulting in shortened root cells, less root meristem tissue, and cell death in the root [62].

*AUXIN RESISTANT 1* (*AUX1*) and *PIN2* regulate root gravitropism. The *aux1-T* mutant shows stronger defects in root gravitropism than the *pin2-T aux1-T* double mutant. The *pin2-T* double mutant shows a similar phenotype to *aux1-T*. No asymmetric distribution of gravity-induced growth hormone response is detectable in the *pin2-T*, *aux1-T*, and *aux1-T pin2-T* mutants. In contrast, the *aux1-T pin2-T* double mutant shows similar growth hormone responses to the *aux1-T* mutant, indicating that *AUX1* controls gravitropic root growth by regulating the asymmetric distribution of growth hormone upstream of *PIN2* [63]. Diallyl disulfide (DADS) has significant effects on tomato seed germination, root growth, mitotic index, root meristem cell size, and hormone content, as well as expression of growth hormone biosynthesis genes (*FZYs*), growth hormone transport genes (*SlPINs*), and expansin genes (*EXPs*) in the root system. Low concentrations (0.01–0.62 mM) of DADS promote root growth, whereas high concentrations (6.20–20.67 mM) have inhibitory effects. The expression levels of *EXP1*, *EXB2*, *EXB3*, *EXLB1*, and β-expanding protein precursors are increased by DADS treatment. These results suggest that tomato root growth is regulated by a variety of expander protein genes at different stages of tomato development [64]. A cell wall-associated receptor kinase (*WAK*) directly links the extracellular matrix to the intracellular compartment and is involved in developmental processes and stress responses. In barley, *HvWAK1* is specifically expressed in the roots. Significant differences in root growth have been observed between wild-type and *HvWAK1*-mutant seedlings of barley under control and salt stress environments [65,66].

### 3.3. Genes Associated with Lateral Root Growth and Development

Root branching is an important factor in root architecture. Lateral roots improve root attachment, water and nutrient uptake, and influence plant growth and development in response to various environmental signals in the soil. Initiation of lateral roots begins with specific mesocolonial sheath cells, that undergo a series of tightly coordinated asymmetric cell divisions to form the lateral root primordia [67]. Initiation of lateral roots is caused by the cyclic death of lateral root cap cells, resulting in a shock to growth hormone signaling in the elongation zone. These cells, characterized by growth hormone signaling, can give rise to a pre-branching site with the ability to form lateral root primordia [68,69]. The formation of the lateral root primordia is characterized by the coordinated nuclear migration of two or three adjacent polar pericycle cells of the xylem, followed by a series of well-organized cell divisions. The involvement of specific molecules is required for different steps of the process before or during lateral root formation [70].

In *Arabidopsis*, *GOLVEN 6* (*GLV6*), a member of the GOLVEN/root growth factor/CLE-like (*GLV/RGF/CLEL*) signal peptide family, is involved in lateral root initiation. Loss-of-function mutation of the related gene *GLV10* results in increased asymmetric cell division during lateral root initiation. The leucine-rich repeat-like receptor kinases RGI1, 4, and 5 may function as recognition receptors for *GLV6* during lateral root initiation. A *GLV6* inhibitor screening revealed that the mitogen-activated protein kinase *MPK6* may function as a direct downstream signal for *GLV6*. *GLV6/10* inhibits asymmetric cell division via the *RGF1*-insensitive receptor and *MPK6* signaling pathway to limit the initial asymmetric cell division that occurs during lateral root initiation. During lateral root initiation, a series of initial asymmetric cell divisions occur [71].

The *LBD* transcription factor is among the most intensively studied downstream target genes of *ARF7*, which is mainly expressed in lateral root primordia and adjacent cell tissues and regulates lateral root development during a novel mechanism for the regulation of lateral root development in *Arabidopsis* [72]. A novel downstream target gene in the ARF7-LBDs-mediated growth hormone signaling pathway, named *PR-1 HOMOLOG 1* (*PRH1*), is involved in the regulation of growth hormone-induced lateral root development in *Arabidopsis* downstream of *ARF7* and *LBDs*. Overexpression of *PRH1* in an *arf7* background partially restored the lateral root phenotype of *ARF7*. *LBDs*, which are also located downstream of *ARF7*, are involved in regulating the transcriptional expression of *PRH1*. Overexpression of *PRH1* partially restored the reduced lateral root phenotype in *lbds* mutants [73,74,75].

Lateral root genesis in *Arabidopsis* begins with the asymmetric division of the guard cell, which undergoes cell proliferation and differentiation to form a new lateral root primordium and eventually new meristematic tissue [76,77]. Genetic and physiological analyses have shown that most developmental events during lateral root formation are regulated by growth hormone signaling [68]. The transcription factors *GATA23* and *LBD16* have been identified as important growth hormone-inducible transcription factors in lateral root formation cells, and *LBD16* can be directly activated by the *SLR*/*IAA14*–*ARF7*–*ARF19* signaling pathway [78,79]. Based on the genetic identification of growth hormone signaling pathways involved in lateral root initiation, *ARF7* activates *LBD16*, which is upstream of the AP2/EREBP-like transcription factor *PUCHI* (a regulator of lateral root primordia) [80,81]. This suggests that spatiotemporal control of *PUCHI* expression by *LBD16* is important for promoting lateral root formation and that initiation of lateral roots requires sequential induction of the *LBD16* and *PUCHI* transcription factors [82].

The adaptation of plant roots to environmental stresses depends largely on the growth and development of lateral roots. In chrysanthemum, *CmANR1*, a homolog of *Arabidopsis AtANR1*, plays a critical role in regulating lateral root development. Ectopic expression of *CmANR1* in *Arabidopsis* significantly increases the number and length of lateral roots compared with the wild type. Moreover, *CmANR1* promotes lateral root growth and development by regulating growth hormone biosynthesis and transport [83]. A novel mechanism has been observed by which *CmANR1* in chrysanthemum can transcriptionally activate expression of the growth hormone transport gene *CmPIN2* and increase the content of growth hormones in the root system, thereby promoting root development. Hydroponically grown transgenic chrysanthemum *CmANR1* has a more extended and stronger root system. In addition, the number and total length of lateral roots and the total volume of the root system are significantly increased compared with wild-type chrysanthemum, suggesting that *CmANR1* may promote lateral root development in chrysanthemum. Furthermore, *CmANR1* promotes lateral root development in chrysanthemum by increasing auxin contents in the root system [84]. In plants, growth hormone distribution is closely associated with lateral root development. Recent research has also shown that auxin activates mitogen-activated protein kinases (*MAPKs*) via transverse membrane kinases (*TMKs*) to control the pattern of cell division during lateral root development. Both *TMK1/4* and *MKK4/5-MPK3/6* signaling pathways are required to control cell division, which ultimately determines lateral root development in response to auxin. In addition, *TMKs* directly and specifically interact with phosphorylate *MKK4/5*, which is required for activation of the *MKK4/5-MPK3/6* pathway by auxin. Thus, TMK-mediated growth hormone signaling promotes lateral root growth and development by regulating cell division patterns via *MAPK* signaling [85].

Maize *deeper rooting 1* (*DRO1*) is negatively regulated by auxin and is involved in the elongation of root tip cells, resulting in asymmetric lateral roots and downward bending under gravity. Overexpression experiments have shown that the higher the expression level of *DRO1*, the greater the angle of lateral root growth and the more the depth of growth [19]. Similarly, *DRO1* influences the direction of lateral root growth. The promoter–reporter structure shows that mutations in *AtDRO1* can lead to greater horizontal entrapment of lateral roots, and overexpression of *PpeDRO1* in plum results in a deeper root phenotype. These data suggest that *DRO1*-related genes play an important role in altering root architecture [86]. In *Arabidopsis*, a family of six genes share five regions of limited sequence similarity with the *LAZY1* gene, and a gene in rice is involved in early gravitropic signaling of lateral root gravitropism. Insertion of T-DNA into the *Arabidopsis* gene *AT5G14090*, which is most similar to *LAZY1*, indicated that *AtLAZY1* is able to regulate the lateral root meristem phenotype in *Arabidopsis* [87].

*KNOTTED-LIKE FROM ARABIDOPSIS THALIANA 1* (*KNAT1*) is involved in regulating the angle of lateral roots in *Arabidopsis* when grown on vertical and inclined agar medium. The two mutant alleles (*bp-1* and *bp-5*) have roots that are excessively inclined to the right side of gravity when grown on a vertical and inclined agar medium surface. Further investigation revealed that the *knat1* mutation significantly reduces growth hormone transport in roots and increases growth hormone accumulation in roots. This change in growth hormone transport is accompanied by a reduction in PIN2 concentration in the root tip, as determined by PIN2-GFP reporter and Western blot analysis. These results suggest that *KNAT1* may negatively affect the gravitropic angle of lateral roots by regulating growth hormone transport [88]. Four rice mutants with different genetic backgrounds show a reduced number of lateral roots and partial loss of gravitropism. Positional cloning of one of the four mutants revealed that this was due to a loss-of-function of the ribosylation factor guanine nucleotide exchange factor (*OsGNOM1*) ADP. In addition, the expression of *OsPIN2*, *OsPIN5b*, and *OsPIN9* is altered in the mutants [89]. *OsIAA3* is an IAA gene family member, for which the expression level increases rapidly in response to growth hormone. Overexpression of *OsIAA3* in rice results in phenotypic traits such as insensitivity to growth hormones and gravitropic stimulation, as well as reduced lateral root formation and abnormal leaf formation [90].

### 3.4. Genes Associated with Root Hair Growth and Development

Root hairs are mainly located in the maturation zone of the root tip and are formed as protrusions from the epidermal cells. Root hairs increase the absorption area of the root and secrete acidic substances that promote the dissolution of salts in soil and thus increase nutrient uptake by the plant [91]. The length of root hairs can vary by as much as 100-fold, and therefore are an excellent system for studying the cellular regulatory mechanisms of the plant root system.

Initiation of root hair growth is regulated by several bHLH transcription factors, such as *Root Hair Defective 6-like 4* (*RSL4*), *RSL2*, and *Root Hair Defective 6* (*RHD6*) [92]. Several growth and developmental regulatory pathways, as well as signals from the growth hormones ethylene and abscisic acid, are important regulators of root hair elongation [93]. In addition, ROS can regulate root hair elongation by oxidizing cell wall-specific components and affecting cell wall cross-linking and hardening [94]. M190905 is located in the intron region of the peroxidase gene *PEROXIDASE 62* (*PRX62*). An additional peroxidase gene, *PRX69*, is highly expressed in root hairs [95]. The FAB1 protein and its product PtdIns (3,5) P2 are localized to the cell membrane of the root hair stalk. Phenotypic analysis after the simultaneous reduction of *FAB1A/FAB1B* expression in artificial microRNA mutants revealed that the root hairs were shorter, wider, wavier, and formed branches. The reduction in *FAB1A/FAB1B* expression also affects the thickness of secondary cell walls in the xylem and the microtubule arrangement in root hairs. Subsequent biochemical experiments have revealed that *RHO-RELATED GTPASES FROM PLANTS 10* (*ROP10*) is involved in sclerotization of the root hair stalk. Furthermore, *ROP10* interacts with *FAB* in root hairs and the cell membrane localization of *ROP10* is dependent on the kinase PtdIns (3,5) P2 [96].

A *zinc finger protein 5* (*zfp5*) mutant and *zfp5* RNAi strains show reduced *ZFP5* function, resulting in fewer and shorter root hairs compared to the wild type. *ZFP5* affects root hair development by directly promoting the expression of *CAPRICE* (*CPC*) [97]. Root hair formation in *Arabidopsis* is mainly controlled by a transcriptional activation complex that induces the homologous gene *GL2* and *MYB* genes with a single repeat *R3* to regulate root hair development. The *gl2* single mutant partially restores the phenotype of a lack of root hairs. The double and higher mutant between *gl2* and a *myb* single mutant have a similar root hair phenotype to the *gl2* single mutant. These results suggest that *gl2* and a single *myb* function in a common pathway to regulate the root hair pattern [98]. Similarly, *WRKY DNA BINDING PROTEIN 75* (*WRKY75*) inhibits the growth and development of *Arabidopsis* root hairs and represses expression of *TRIPTYCHON* and *CPC*. A yeast one-hybrid assay showed that the WRKY75 protein bound to the CPC promoter. The *WRKY75* gene is mainly expressed in the mesocolonial sheath and vascular tissue [99]. In addition, *Arabidopsis* mutants with an altered root hair phenotype have been used to study cell wall dynamics and the expression of S-Nitrosoglutathione (*GSNO*) in roots during the induction of root hair formation. *GSNO* and growth hormone were jointly involved in the restoration of the root hair phenotype in the root-hairless *rhd6* mutant. *GSNO* regulated the expression of a large number of genes associated with cell wall composition and metabolism, as well as genes encoding ribosomal proteins, DNA- and histone-modifying enzymes, and proteins involved in post-translational modifications [100].

In the rice mutant *rth1*, root hair elongation is eliminated after swelling. Sequence comparison of three genes from the wild type and the *rth1* mutant revealed a nucleotide substitution in *OsAPY* only. This nucleotide substitution results in abnormal splicing of the cDNA sequence in the *rth1* mutant. Following introduction of the *OsAPY* allele, the transgenic plants developed normal root hairs and showed the complementary phenotype of the *rth1* mutant. This suggests that *OsAPY* may directly regulate root hair growth and development in rice [101]. The cellulose synthase-like gene *OsCSLD1* is required for root hair growth and development, and rice mutants defective for *OsCSLD1* exhibit an abnormal root hair phenotype or lack root hairs. Gene expression analysis and an in situ hybridization analysis showed that *OsCSLD1* is expressed only in root hair cells. *OsCSLD*1 is the only gene among four *OsCSLD* genes that exhibits root-specific expression [102]. Yuo et al. demonstrated the molecular mechanisms involved in the specific expression of *OsCSLD1* in root hairs and the growth and development of rice root hairs [103]. A rice T-DNA mutant with short root hairs, designated as *ossrh3* (*oryza sativa short root hair 3*), exhibits severely impaired root hair elongation together with changes in plant height, main root length, lateral root length, and number of lateral roots. Genetic analysis showed that the mutant trait is controlled by a single pair of recessive genes, and *OsSRH3* has been identified by molecular marker and localization analyses to be responsible for root hair development in rice [104,105,106].

A gene encoding EXPANSIN A17 (EXPA17) was identified in a rice mutant with short root hairs. The mutant OsEXPA17 protein contains a point mutation that results in a change in amino acid sequence. Further studies in which *OsEXPA17* expression was inhibited by RNAi confirmed that *OsEXPA17* was capable of regulating root hair elongation in rice. Complementation of the *OsEXPA17* mutant with the root hair-expressed genes *OsEXPA30* and *AtEXPA7* in *Arabidopsis* restored root hair elongation, suggesting that members of the root hair expansion protein subclass play a crucial role in root hair formation [107,108]. In maize, root hairs of the recessive root-hairless mutant *rth6* bulged at the transition to root tip growth, and then stopped growing and exhibited a rough cell surface. A phylogenetic analysis showed that *ROOTHAIRLESS 6* (*RTH6*) belongs to the D-type cellulose synthase branch, which is found only in monocotyledons, and that D-type cellulose synthase is highly conserved in the plant kingdom, with five gene family members in maize. Expression profiling showed that *RTH6* is highly enriched in root hairs compared with other root tissues [109].

## 4. Conclusions

Declining crop yields caused by abiotic stresses pose a major challenge to the production of staple crops, and global demand for food is expected to exceed genetic advances in the near future. Thus, environmental changes pose a significant risk to food security. Therefore, integrated solutions are needed to address these challenges and increase crop yields to ensure food security. Plant roots are the first organs to sense drought stress and their morphological structure plays a pivotal role in coping with drought stress [27,110]. When plants are under drought stress, the number of root branches and root hairs increases as the root system becomes more deeply rooted, maximizing the uptake and utilization of nutrients and water in the soil. Therefore, plant root architecture is an important phenotypic indicator of drought resistance in crops [111]. In recent years, studies on the root system of tuber crops have shown that root architecture is strongly correlated with crop yield [112,113]. Exploring the relationship between root architecture and abiotic interaction holds great potential to provide answers to pressing problems, such as root plasticity and crop improvement. As a typical shallow-rooted tuber crop, potato roots penetrate the soil poorly and are sensitive to drought throughout the growing season [114]. The improvement of root architecture is an important direction in breeding potato for drought stress resistance because it can promote faster growth rates, smaller angles of lateral root branches and an increase in the number of root hairs. Thus, a greater number of lateral roots and root hairs can penetrate deeper into the soil to absorb water from lower soil layers under drought stress. Therefore, it is important to explore genes involved in root development in model plants and other crops, such as rice and maize, to identify and screen their homologs in potato to perform functional validations and in-depth studies to improve the root architecture of potato.

Investigation of the root development-related genes discussed in this review will enrich our understanding of root architecture establishment and abiotic stress resistance and expand our knowledge of gene function in improving plant productivity. Breeding stress-resistant plants with high yields will lead to crop improvement. Elucidating the molecular mechanisms of root development genes will provide insights into the different genes and their specific roles in adaptation to various abiotic stresses, thereby providing a better understanding of the targeted opportunities for crop improvement and drought tolerance breeding.

## Figures and Tables

**Table 1:** Genes related to the development of different parts of plant roots.

Root Type	Plant Species	Genes
Adventitious root	*Arabidopsis thaliana*	*PIN1/3, ARF7/17/19, MDR1, SHR, SOS3, WOX5/12*
	*Oryza sativa* L.	*PIN1/2, RAA1, AGAP, GNOM1, MT2b, NAL1, PIN3t, RCC3, CRL1, CAND1, LOB16, CKX4, IAA3, TIR1, WOX11*
	*Zea mays* L.	*RTCL1, RTCS1, LOB6, RTCS*
	*Solanum lycopersicum*	*CCD8*
	*Capsicum annuum* L.	*CaNAC46, CaDSR6*
	*Gossypium hirsutum*	*PIN2*
Lateral root	*Arabidopsis thaliana*	*GLV6/10, ARF7/19, EXP4, ASA1, GATA23, PIN3, SCR, PLT1/2, BRX, PYL8, ALF4, DRO1, LAZY1, KNAT1, ABI3/4, AFB2/3, AGL21/44, ALF1/3/4, ARF7/8/19, AUX1, AXR1/4, BARK1, CEG, CRF2/3, DFL1, E2FA, EIR1, ETR1, FUS3, GNOM, IAA18/19/28, IQM3, KNAT1/3, KRP1/2, LAX3, LBD13/14, LEC2, MDR1, MIZ1, MKK6, MPK13, MUL, MUS, MYB44, NAC1/2, PGP1/4, PHB, PHV, PIN3/7, PLC5, PRE3, PUCHI, PYL8/9, REV, RML2, SGT1B, SHR, SOS3, SWP1, TIR1,WOX7/9, WRKY46, XBAT32, YUCCA4*
	*Oryza sativa* L.	*GNOM, CYP2, ORC3, MT2b, AUX1, NAC9/10, IAA3/11/13, LAZY1, DRO1, CML16, EXPA8, GRXC12, MADS25, NAR2.1, RCc3, WRKY28*
	*Zea mays* L.	*NAC1, Rum1, ARF34, LA1, IRT1, SLR1/2*
	*Gossypium hirsutum*	*NAC9, EXP4*
	*Glycine max*	*LBD12, WRKY13, NAC1, EXPB2, WNK1*
	*Triticum aestivum* L.	*WRKY51*
	*Solanum lycopersicum*	*EXP4, IAA7, ARF2, DGT, MBP9*
Primary root	*Arabidopsis thaliana*	*KNAT5, HAP3b, DGL1, MAIN, MAIL, RGS1, RGF1, PERK4, ABA2, AFB3, AGL12/14, ARF2, CKX7, DWF4, EIN3, ERF1, GNOM, HYD1, IQM3, MAIL1, MED12, MRP5, PERK4/8, PIN1/2, PLC5, RML1/2, SHR, WOX9/14, UPB1, BSK3*
	*Oryza sativa* L.	*GNA1, GLU3, SPR1, PIN3t, AGAP, ARF12, GLR3.1, DGL1, SOR1, AGAP, AKT1, CML16, CRL2, DGL1, EXPA8, MADS25, MOGS, RAA1, RCc3, RRL1/2*
	*Zea mays* L.	*AUX1, EXPB2*
	*Solanum lycopersicum*	*EXP1, EXPB2*
	*Glycine max*	*WAK1*
Root hair	*Arabidopsis thaliana*	*COW1, RHD6, IAA7, CTR1, BRI1, AKT1, AXR2, COW1, CPC, CPL3, ETC1/2, EXPA7, FH8, GL1, GLV4, HDG11, IAA17, KOJAK, LRX1/2, MED12/13, MRH1, PERK13, PGP4, PLC5, PRP3, RHD1, ROP2, SOS4, TIP1, TRH1, TTG, WRKY75, ZFP5*
	*Oryza sativa* L.	*APY2, CSLD1, EXPB5, RHL1, SRH1/3, EXP17, YUCCA1, EXPA30, FH1, NOX3, XXT1*
	*Zea mays* L.	*RTH1/2*
	*Hordeum vulgare* L.	*EXPB1/7*
	*Gossypium hirsutum*	*EXPA4*
	*Triticum aestivum* L.	*RSL2/4*
	*Solanum lycopersicum*	*LKT1, SlTRY, SlGL3*
	*Glycine max*	*EXPB2*

## Data Availability

Not applicable.

## References

[1] Gleick P.H. (2002). The World’s Water, 2000–2001, The Biennial Report on Freshwater Resources. Electron. Green J..

[2] FAO (2019). Proactive Approaches to Drought Preparedness—Where Are We Now and Where do We Go from Here?.

[3] Gupta A., Rico-Medina A., Caño-Delgado A.I. (2020). The physiology of plant responses to drought. Science.

[4] Koncagül E., Tran M., Connor S., Uhlenbrook S. (2018). World Water Development Report 2020—Water and Climate Change (SC-2018/WS/5, UNESCO WWAP).

[5] Xoconostlecázares B., Ramírezortega F., Floreselenes L., Ruizmedrano R. (2010). Drought Tolerance in Crop Plants. Am. J. Plant Physiol..

[6] Supratim B., Venkategowda R., Anuj K., Pereira A. (2016). Plant adaptation to drought stress. F1000 Res..

[7] Tardieu F., Simonneau T., Muller B. (2018). The Physiological Basis of Drought Tolerance in Crop Plants, A Scenario-Dependent Probabilistic Approach. Annu. Rev. Plant Biol..

[8] Millet E.J., Welcker C., Kruijer W., Negro S., Coupel-Ledru A., Nicolas S.D., Laborde J., Bauland C., Praud S., Ranc N. (2016). Genome-Wide Analysis of Yield in Europe, Allelic Effects Vary with Drought and Heat Scenarios. Plant Physiol..

[9] Tuberosa R., Salvi S. (2006). Genomics approaches to improve drought tolerance in crops. Trends Plant Sci..

[10] Ashraf M., Akram N.A. (2009). Improving salinity tolerance of plants through conventional breeding and genetic engineering: An analytical comparison. Biotechnol. Adv..

[11] Ahmed K., Shabbir G., Ahmed M., Shah K.N. (2020). Phenotyping for drought resistance in bread wheat using physiological and biochemical traits. Sci. Total Environ..

[12] Hardigan M.A., Laimbeer F.P.E., Newton L., Crisovan E., Hamilton J.P., Vaillancourt B., Wiegert-Rininger K., Wood J.C., Douches D.S., Farré E.M. (2017). Genome diversity of tuber-bearing *Solanum* uncovers complex evolutionary history and targets of domestication in the cultivated potato. Proc. Natl. Acad. Sci. USA.

[13] Li Y., Colleoni C., Zhang J., Liang Q., Hu Y., Ruess H., Simon R., Liu Y., Liu H., Yu G. (2018). Genomic Analyses Yield Markers for Identifying Agronomically Important Genes in Potato. Mol. Plant.

[14] Sun C., Liang W., Yan K., Xu D., Qin T., Fiaz S., Kear P., Bi Z., Liu Y., Liu Z. (2022). Expression of Potato StDRO1 in *Arabidopsis* Alters Root Architecture and Drought Tolerance. Front. Plant Sci..

[15] Martínez I., Muñoz M., Acuña I., Uribe M. (2021). Evaluating the Drought Tolerance of Seven Potato Varieties on Volcanic Ash Soils in a Medium-Term Trial. Front. Plant Sci..

[16] Guo W., Chen L., Herrera-Estrella L., Cao D., Tran L.-S.P. (2020). Altering Plant Architecture to Improve Performance and Resistance. Trends Plant Sci..

[17] Zhang H., Zhu J., Gong Z., Zhu J.-K. (2022). Abiotic stress responses in plants. Nat. Rev. Genet..

[18] Ramachandran P., Augstein F., Nguyen V., Carlsbecker A. (2020). Coping with Water Limitation: Hormones That Modify Plant Root Xylem Development. Front Plant Sci..

[19] Steffens B., Wang J., Sauter M. (2006). Interactions between ethylene, gibberellin and abscisic acid regulate emergence and growth rate of adventitious roots in deepwater rice. Planta.

[20] Uga Y., Sugimoto K., Ogawa S., Rane J., Ishitani M., Hara N., Kitomi Y., Inukai Y., Ono K., Kanno N. (2013). Control of root system architecture by DEEPER ROOTING 1 increases rice yield under drought conditions. Nat. Genet..

[21] Caldeira C.F., Jeanguenin L., Chaumont F. (2014). Circadian rhythms of hydraulic conductance and growth are enhanced by drought and improve plant performance. Nat. Commun..

[22] Orman-Ligeza B., Morris E.C., Parizot B., Lavigne T., Babé A., Ligeza A., Klein S., Sturrock C., Xuan W., Novák O. (2018). The Xerobranching Response Represses Lateral Root Formation When Roots Are not in Contact with Water. Curr. Biol..

[23] Maurel C., Nacry P. (2020). Root architecture and hydraulics converge for acclimation to changing water availability. Nat. Plants.

[24] Zia R., Nawaz M.S., Siddique M.J., Hakim S., Imran A. (2021). Plant survival under drought stress: Implications, adaptive responses, and integrated rhizosphere management strategy for stress mitigation. Microbiol. Res..

[25] Andrés F., Coupland G. (2012). The genetic basis of flowering responses to seasonal cues. Nat. Rev. Genet..

[26] Rellán-Álvarez R., Lobet G., Dinneny J.R. (2016). Environmental Control of Root System Biology. Annu. Rev. Plant Biol..

[27] Dietrich D., Pang L., Kobayashi A., Fozard J.A., Boudolf V., Bhosale R., Antoni R., Nguyen T., Hiratsuka S., Fujii N. (2017). Root hydrotropism is controlled via a cortex-specific growth mechanism. Nat. Plants.

[28] Scharwies J.D., Dinneny J.R. (2019). Water transport, perception, and response in plants. J. Plant Res..

[29] Buckley T.N. (2019). How do stomata respond to water status. New Phytol..

[30] Qaseem M., Qureshi R., Shaheen H. (2019). Effects of Pre-Anthesis Drought, Heat and Their Combination on the Growth, Yield and Physiology of diverse Wheat (*Triticum aestivum* L.) Genotypes Varying in Sensitivity to Heat and drought stress. Sci. Rep..

[31] Di Gioia F., Aprile A., Sabella E., Santamaria P., Pardossi A., Miceli A., De Bellis L., Nutricati E. (2017). Grafting response to excess boron and expression analysis of genes coding boron transporters in tomato. Plant Biol..

[32] Schumacher C., Thümecke S., Schilling F., Köhl K., Kopka J., Sprenger H., Hincha D.K., Walther D., Seddig S., Peters R. (2021). Genome-Wide Approach to Identify Quantitative Trait Loci for Drought Tolerance in Tetraploid Potato (*Solanum tuberosum* L.). Int. J. Mol. Sci..

[33] Potocka I., Szymanowska-Pulka J. (2018). Morphological responses of plant roots to mechanical stress. Ann. Bot..

[34] Jing H., Strader L.C. (2019). Interplay of Auxin and Cytokinin in Lateral Root Development. Int. J. Mol. Sci..

[35] Kikuchi A., Huynh H.D., Endo T., Watanabe K. (2015). Review of recent transgenic studies on abiotic stress tolerance and future molecular breeding in potato. Breed Sci..

[36] Achar D., Awati M.G., Udayakumar M., Prasad T.G. (2015). Identification of Putative Molecular Markers Associated with Root Traits in Coffea canephora Pierre ex Froehner. Mol. Biol. Int..

[37] Joshi M., Fogelman E., Belausov E., Ginzberg I. (2016). Potato root system development and factors that determine its architecture. J. Plant Physiol..

[38] Berendsen R.L., Pieterse C.M.J., Bakker P.A.H.M. (2012). The rhizosphere microbiome and plant health. Trends Plant Sci..

[39] Motte H., Vanneste S., Beeckman T. (2019). Molecular and Environmental Regulation of Root Development. Annu Rev. Plant Biol..

[40] Zhao Y., Hu Y., Dai M., Huang L., Zhou D.-X. (2009). The WUSCHEL-Related Homeobox Gene WOX11 Is Required to Activate Shoot-Borne Crown Root Development in Rice. Plant Cell.

[41] Zhao Y., Cheng S., Song Y., Huang Y., Zhou S., Lui Z., Zhou D.-X. (2015). The Interaction between Rice ERF3 and WOX11 Promotes Crown Root Development by Regulating Gene Expression Involved in Cytokinin Signaling. Plant Cell.

[42] Xu C., Tai H., Saleem M., Ludwig Y., Majer C., Berendzen K., Nagel K.A., Wojciechowski T., Meeley R.B., Taramino G. (2015). Cooperative action of the paralogous maize lateral organ boundaries (LOB) domain proteins RTCS and RTCL in shoot-borne root formation. New Phytol..

[43] Blilou I., Xu J., Wildwater M., Willemsen V., Paponov I., Friml J., Heidstra R., Aida M., Palme K., Scheres B. (2005). The PIN auxin efflux facilitator network controls growth and patterning in *Arabidopsis* roots. Nature.

[44] Feraru E., Feraru M.I., Kleine-Vehn J., Martinière A., Mouille G., Vanneste S., Vernhettes S., Runions J., Friml J. (2011). PIN polarity maintenance by the cell wall in *Arabidopsis*. Curr. Biol..

[45] Xu M., Zhu L., Shou H., Wu P.A. (2005). PIN1 family gene, OsPIN1, involved in auxin-dependent adventitious root emergence and tillering in rice. Plant Cell Physiol..

[46] Zhu J., Li Y., Lin J., Wu Y., Guo H., Shao Y., Wang F., Wang X., Mo X., Zheng S. (2019). CRD1, an Xpo1 domain protein, regulates miRNA accumulation and crown root development in rice. Plant J..

[47] Bari V.K., Nassar J.A., Kheredin S.M., Gal-On A., Ron M., Britt A., Steele D., Yoder J., Aly R. (2019). CRISPR/Cas9-mediated mutagenesis of CAROTENOID CLEAVAGE DIOXYGENASE 8 in tomato provides resistance against the parasitic weed Phelipanche aegyptiaca. Sci. Rep..

[48] Lahlou O., Ledent J.-F. (2005). Root mass and depth, stolons and roots formed on stolons in four cultivars of potato under water stress. Eur. J. Agron..

[49] Joshi M., Ginzberg I. (2021). Adventitious root formation in crops-Potato as an example. Physiol. Plant.

[50] Jia Z., Giehl R.F.H., Meyer R.C., Altmann T., von Wiren N. (2019). Natural variation of BSK3 tunes brassinosteroid signaling to regulate root foraging under low nitrogen. Nat. Commun..

[51] Tsukagoshi H., Busch W., Benfey P.N. (2010). Transcriptional regulation of ROS controls transition from proliferation to differentiation in the root. Cell.

[52] Ballif J., Endo S., Kotani M., MacAdam J., Wu Y. (2011). Over-expression of HAP3b enhances primary root elongation in *Arabidopsis*. Plant Physiol. Biochem..

[53] Luxán-Hernández C., Lohmann J., Hellmeyer W., Seanpong S., Wöltje K., Magyar Z., Pettkó-Szandtner A., Pélissier T., De Jaeger G., Hoth S. (2020). PP7L is essential for MAIL1-mediated transposable element silencing and primary root growth. Plant J..

[54] Ühlken C., Horvath B., Stadler R., Sauer N., Weingartner M. (2014). MAIN-LIKE1 is a crucial factor for correct cell division and differentiation in *Arabidopsis thaliana*. Plant J..

[55] Ou Y., Lu X., Zi Q., Xun Q., Zhang J., Wu Y., Shi H., Wei Z., Zhao B., Zhang X. (2016). RGF1 INSENSITIVE 1 to 5, a group of LRR receptor-like kinases, are essential for the perception of root meristem growth factor 1 in *Arabidopsis thaliana*. Cell Res..

[56] Inukai Y., Miwa M., Nagato Y., Kitano H., Yamauchi A. (2001). RRL1, RRL2 and CRL2 loci regulating root elogation in rice. Breed. Sci..

[57] Sun C., Li D., Gao Z., Gao L., Shang L., Wang M., Qiao J., Ding S., Li C., Geisler M. (2022). OsRLR4 binds to the OsAUX1 promoter to negatively regulate primary root development in rice. J. Integr. Plant Biol..

[58] Zhuang X., Xu Y., Chong K., Lan L., Xu Z., Xue Y. (2010). OsAGAP, an ARF-GAP from rice, regulates root development mediated by auxin in *Arabidopsis*. Plant Cell Environ..

[59] Li J., Zhu S., Song X., Shen Y., Chen H., Yu J., Yi K., Liu Y., Karplus V.J., Wu P. (2006). A rice glutamate receptor-like gene is critical for the division and survival of individual cells in the root apical meristem. Plant Cell.

[60] Jia L., Wu Z., Hao X., Carrie C., Zheng L., Whelan J., Wu Y., Wang S., Wu P., Mao C. (2011). Identification of a novel mitochondrial protein, short postembryonic roots 1 (SPR1), involved in root development and iron homeostasis in *Oryza sativa*. New Phytol..

[61] Zhang J.-W., Xu L., Wu Y.-R., Chen X.-A., Liu Y., Zhu S.-H., Ding W.-N., Wu P., Yi K.-K. (2012). OsGLU3, a Putative Membrane-Bound Endo-1,4-Beta-Glucanase, Is Required for Root Cell Elongation and Division in Rice (*Oryza sativa* L.). Mol. Plant.

[62] Cheng Q., Li Y., Jian G., Wang W., Zhang H., Lui Y., Wu P. (2013). OsDGL1, a Homolog of an Oligosaccharyltransferase Complex Subunit, is Involved in N-Glycosylation and Root Development in Rice. Plant Cell Physiol..

[63] Liu H., Liu B., Chen X., Zhu H., Zou C., Men S. (2018). AUX1 acts upstream of PIN2 in regulating root gravitropism. Biochem. Biophys. Res. Commun..

[64] Fang C., Zhihui C., Huanwen M., Tang X. (2016). The Garlic Allelochemical Diallyl Disulfide Affects Tomato Root Growth by Influencing Cell Division, Phytohormone Balance and Expansin Gene Expression. Front. Plant Sci..

[65] Kaur R., Singh K., Singh J. (2013). A root-specific wall-associated kinase gene, HvWAK1, regulates root growth and is highly divergent in barley and other cereals. Funct. Integr. Genom..

[66] Tripathi R., Aguirre J., Singh J. (2021). Genome-wide analysis of wall associated kinase (WAK) gene family in barley. Genomics.

[67] Moreno-Risueno M.A., Van Norman J.M., Moreno A., Zhang J., Ahnert S.E., Benfey P.N. (2010). Oscillating gene expression determines competence for periodic *Arabidopsis* root branching. Science.

[68] Xuan W., Band L.R., Kumpf R.P., Van Damme D., Parizot B., De Rop G., Opdenacker D., Möller B.K., Skorzinski N., Njo M.F. (2016). Cyclic programmed cell death stimulates hormone signaling and root development in *Arabidopsis*. Science.

[69] De Smet I., Lau S., Voß U., Vanneste S., Benjamins R., Rademacher E.H., Schlereth A., De Rybel B., Vassileva V., Grunewald W. (2010). Bimodular auxin response controls organogenesis in *Arabidopsis*. Proc. Natl. Acad. Sci. USA.

[70] De Ryble B., Vassileva V., Parizot B., Demeulenaere M., Grunewald W., Audenaert D., Van Campenhout J., Overvoorde P., Jansen L., Vanneste S. (2010). A novel aux/IAA28 signaling cascade activates GATA23-dependent specification of lateral root founder cell identity. Curr. Biol..

[71] Fernandez A.I., Vangheluwe N., Xu K., Jourquin J., Claus L.A.N., Morales S., Parizot B., De Gernier H., Yu Q., Drozdzecki A. (2020). GOLVEN peptide signalling through RGI receptors and MPK6 restricts asymmetric cell division during lateral root initiation. Nat. Plants.

[72] Huang K.-L., Ma G.-J., Zhang M.-L., Xiong H., Wu H., Zhao C.-Z., Liu C.-S., Jia H.-X., Chen L., Kjorven J.O. (2018). The ARF7 and ARF19 Transcription Factors Positively Regulate PHOSPHATE STARVATION RESPONSE1 in *Arabidopsis* Roots. Plant Physiol..

[73] Yu J., Xie Q., Li C., Dong Y., Zhu S., Chen J. (2020). Comprehensive characterization and gene expression patterns of LBD gene family in *Gossypium*. Planta.

[74] Wang J., Zhang W., Cheng Y., Feng L. (2021). Genome-Wide Identification of LATERAL ORGAN BOUNDARIES DOMAIN (LBD) Transcription Factors and Screening of Salt Stress Candidates of *Rosa rugosa* Thunb. Biology.

[75] Zhang F., Tao W., Sun R., Wang J., Li C., Kong X., Tian H., Ding Z. (2022). Correction: PRH1 mediates ARF7-LBD dependent auxin signaling to regulate lateral root development in *Arabidopsis thaliana*. PLoS Genet..

[76] Goh T., Toyokura K., Wells D.M., Swarup K., Yamamoto M., Mimura T., Weijers D., Fukaki H., Laplaze L., Bennett M.J. (2016). Quiescent center initiation in the *Arabidopsis* lateral root primordia is dependent on the SCARECROW transcription factor. Development.

[77] Wangenheim D., Fangerau J., Schmitz A., Smith R., Leitte H., Stelzer E.H., Maizel A. (2016). Rules and self-organizing properties of post-embryonic plant organ cell division patterns. Curr. Biol..

[78] Okushima Y., Fukaki H., Onoda M., Theologis A., Tasaka M. (2007). ARF7 and ARF19 regulate lateral root formation via direct activation of LBD/ASL genes in *Arabidopsis*. Plant Cell.

[79] Goh T., Joi S., Mimura T., Fukaki H. (2012). The establishment of asymmetry in *Arabidopsis* lateral root founder cells is regulated by LBD16/ASL18 and related LBD/ASL proteins. Development.

[80] Hirota A., Kato T., Fukaki H., Aida M., Tasaka M. (2007). The auxin-regulated AP2/EREBP gene *PUCHI* is required for morphogenesis in the early lateral root primordium of *Arabidopsis*. Plant Cell.

[81] Lavenus J., Goh T., Guyomarc’H S., Hill K., Lucas M., Voss U., Kenobi K., Wilson M., Farcot E., Hagen G. (2015). Inference of the *Arabidopsis* lateral root gene regulatory network suggests a bifurcation mechanism that defines primordia flanking and central zones. Plant Cell.

[82] Goh T., Toyokura K., Yamaguchi N., Okamoto Y., Uehara T., Kaneko S., Takebayashi Y., Kasahara H., Ikeyama Y., Okashima Y. (2019). Lateral root initiation requires the sequential induction of transcription factors LBD16 and PUCHI in *Arabidopsis thaliana*. New Phytol..

[83] Sun C.-H., Yu J.-Q., Wen L.-Z., Guo Y.-H., Sun X., Hao Y.-J., Hu D.-G., Zheng C.-H. (2018). Chrysanthemum MADS-box transcription factor CmANR1 modulates lateral root development via homo-/heterodimerization to influence auxin accumulation in *Arabidopsis*. Plant Sci..

[84] Sun C.-H., Yu J.-Q., Duan X., Wang J.-H., Zhang Q.-Y., Gu K.-D., Hu D.-G., Zheng C.-S. (2018). The MADS transcription factor CmANR1 positively modulates root system development by directly regulating CmPIN2 in chrysanthemum. Hortic Res..

[85] Huang R., Zheng R., He J., Zhou Z., Wang J., Xiong Y., Xu T. (2019). Noncanonical auxin signaling regulates cell division pattern during lateral root development. Proc. Natl. Acad. Sci. USA.

[86] Guseman J., Webb K., Srinivasan C., Dardick C. (2017). *DRO1* influences root system architecture in *Arabidopsis* and Prunus species. Plant J..

[87] Yoshihara T., Spalding E., Iino M. (2013). AtLAZY1 is a signaling component required for gravitropism of the *Arabidopsis thaliana* inflorescence. Plant J..

[88] Bin Q., Huiqiong Z. (2013). Modulation of root-skewing responses by KNAT1 in *Arabidopsis thaliana*. Plant J..

[89] Liu S., Wang J., Wang L., Wang X., Xue Y., Wu P., Shou H. (2009). Adventitious root formation in rice requires OsGNOM1 and is mediated by the OsPINs family. Cell Res..

[90] Nakamura A., Umemura I., Gomi K., Hasegawa Y., Kitano H., Sazuka T. (2010). Production and characterization of auxin-insensitive rice by overexpression of a mutagenized rice IAA protein. Plant J..

[91] Mangano S., Denita-Juarez S., Marzol E., Borassi C., Estevez J.M. (2018). High Auxin and High Phosphate Impact on RSL2 Expression and ROS-Homeostasis Linked to Root Hair Growth in *Arabidopsis thaliana*. Front. Plant Sci..

[92] Datta S., Prescott H., Dolan L. (2015). Intensity of a pulse of RSL4 transcription factor synthesis determines *Arabidopsis* root hair cell size. Nat. Plants.

[93] Zhang S., Huang L., Yan A., Liu Y., Liu B., Yu C., Zhang A., Schiefelbein J., Gan Y. (2016). Multiple phytohormones promote root hair elongation by regulating a similar set of genes in the root epidermis in *Arabidopsis*. J. Exp. Bot..

[94] Bhosale R., Giri J., Pandey B.K., Giehl R.F.H., Hartmann A., Traini R., Truskina J., Leftley N., Hanlon M., Swarup K. (2018). A mechanistic framework for auxin dependent *Arabidopsis* root hair elongation to low external phosphate. Nat. Commun..

[95] Pacheco J.M., Ranocha P., Kasulin L., Fusari C.M., Servi L., Aptekmann A.A., Gabarain V.B., Peralta J.M., Borassi C., Marzol E. (2022). Apoplastic class III peroxidases PRX62 and PRX69 promote *Arabidopsis* root hair growth at low temperature. Nat. Commun..

[96] Hirano T., Konno H., Takeda S., Dolan L., Kato M., Aoyama T., Higaki T., Takigawa-Imamura H., Sato M.H. (2018). PtdIns (3,5) P2 mediates root hair shank hardening in *Arabidopsis*. Nat. Plants.

[97] An L., Zhou Z., Sun L., Yan A., Xi W., Yu N., Cai W., Chen X., Yu H., Schiefelbein J. (2012). A zinc finger protein gene ZFP5 integrates phytohormone signaling to control root hair development in *Arabidopsis*. Plant J..

[98] Wang S., Barron C., Schiefelbein J., Chen J.-G. (2010). Distinct relationships between GLABRA2 and single-repeat R3 MYB transcription factors in the regulation of trichome and root hair patterning in *Arabidopsis*. New Phytologist.

[99] Rishmawi L., Pesch M., Juengst C., Schauss A.C., Schrader A., Hülskamp M. (2014). Non-Cell-Autonomous Regulation of Root Hair Patterning Genes by WRKY75 in *Arabidopsis*. Plant Physiol..

[100] Moro C., Gaspar M., Silva F., Pattathil S., Hahn M., Salgado I., Braga M.R. (2017). S-nitrosoglutathione promotes cell wall remodelling, alters the transcriptional profile and induces root hair formation in the hairless *root hair defective 6* (*rhd6*) mutant of *Arabidopsis thaliana*. New Phytol..

[101] Takahisa Y., Masanori T., Masahiko I., Taketa S. (2009). Molecular cloning of a root hairless gene rth1 in rice. Breed. Sci..

[102] Kim C.M., Park S.H., Je B.I., Park S.H., Park S.J., Piao H.L., Eun M.Y., Dolan L., Han C.-D. (2007). OsCSLD1, a cellulose synthase-like D1 gene, is required for root hair morphogenesis in rice. Plant Physiol..

[103] You T., Shiotani K., Shitsukawa N., Miyao A., Hirochika H., Ichii M., Taketa S. (2012). Root hairless 2 (rth2) mutant represents a loss-of-function allele of the cellulose synthase-like gene OsCSLD1 in rice (*Oryza sativa* L.). Breed. Sci..

[104] Ding W.-N., Huang W., Ning Y.-Q., Zhu S.-H. (2012). Genetic Analysis and Mapping of a Novel Short Root Hair Gene OsSRH3 in Rice. Acta Agron. Sin..

[105] Ding W., Tong Y., Ning Y., Zhu S. (2011). Phenotypic analysis and gene mapping of a short root hair mutant Ossrh2 in rice (*Oryza sativa*). Chin. Bull. Bot..

[106] Ding W., Tong Y., Wu J., Zhu S. (2011). Identification and gene mapping of a novel short root hair mutant in rice. Sci. Agric. Sin..

[107] Won S., Kumari S., Choi S., Cho M., Lee S., Cho H. (2010). Root hair-specific EXPANSIN B genes have been selected for Graminaceae root hairs. Mol. Cells.

[108] Yu Z., Bo K., He X., Lv S., Bai Y., Ding W., Chen M., Cho H.-T., Wu P. (2011). Root hair-specific expansins modulate root hair elongation in rice. Plant J..

[109] Li L., Hey S., Liu S., Liu Q., McNinch C., Hu H.-C., Wen T.-J., Marcon C., Paschold A., Bruce W. (2016). Characterization of maize roothairless6 which encodes a D-type cellulose synthase and controls the switch from bulge formation to tip growth. Sci. Rep..

[110] Carminati A., Vetterlein D. (2013). Plasticity of rhizosphere hydraulic properties as a key for efficient utilization of scarce resources. Ann. Bot..

[111] Nardini A., Casolo V., Borgo A., Savi T., Stenni B., Bertoncin P., Zini L., McDowell N.G. (2016). Rooting depth, water relations and non-structural carbohydrate dynamics in three woody angiosperms differentially affected by an extreme summer drought. Plant Cell Environ..

[112] Griffiths M., York L. (2020). Targeting Root Ion Uptake Kinetics to Increase Plant Productivity and Nutrient Use Efficiency. Plant Physiol..

[113] de Vries F.T., Griffiths R.I., Knight C.G., Nicolitch O., Williams A. (2020). Harnessing rhizosphere microbiomes for drought-resilient crop production. Science.

[114] Gervais T., Creelman A., Li X.-Q., Bizimungu B., De Koeyer D., Dahal K. (2021). Potato Response to Drought Stress: Physiological and Growth Basis. Front. Plant Sci..

